# Global Awareness and Response to Early Symptoms of Acute Stroke: A Systematic Literature Review

**DOI:** 10.7759/cureus.78420

**Published:** 2025-02-03

**Authors:** Theodoros Vatsalis, Dimitrios Papadopoulos, Vasiliki Georgousopoulou, Prodromos Bostantzis, Jobst Rudolf

**Affiliations:** 1 Cardiology, International Hellenic University, Drama, GRC; 2 Intensive Care Unit, General Hospital Of Larisa, Larisa, GRC; 3 Internal Medicine, University Hospital Of Alexandroupoli, Alexandroupoli, GRC; 4 Internal Medicine, General Hospital of Kavala, Kavala, GRC; 5 Neurology, General Hospital of Τhessaloniki Papageorgiou, Thessaloniki, GRC

**Keywords:** awareness, early symptoms, response, s: knowledge, stroke

## Abstract

Stroke is a condition that leaves persistent disability and causes high mortality worldwide. Knowledge of recognizing early symptoms along with awareness of immediate response to acute stroke among the general population has proven insufficient. Pre-attendance delays adversely affect the time window from symptoms onset to needle time, therefore the effect of recanalization treatments. Although 35% of patients are potentially eligible for these treatments such as intravenous thrombolysis only about about 8-10% of them manage to receive. The study aimed to investigate the association between knowledge of early symptoms in the general population and proper response to acute stroke through prompt activation of emergency medical services (EMS) independently of sociodemographic factors. A systematic review was conducted to identify relevant studies that assessed the general population’s knowledge of early symptom recognition and awareness of immediate response to acute stroke. Two investigators reviewed articles published in the PubMed database, from December 1, 2023, to February 29, 2024. The search yielded a total of 340 articles. After the 340 articles were screened, 293 reports were excluded due to title and abstract, irrelevant studies, or when the full text was not available. Finally, a total of 10 articles were included in this systematic review. Out of ten studies included in this review, seven studies were conducted in Asia, in one study the origin continent is Oceania, while the remaining two studies come from America.

All studies were designed and conducted as cross-sectional studies. The most commonly recognizable early symptoms of a stroke were found to be difficulty in speaking or understanding speech, weakness on one side of the body including the face, plus sudden dizziness. Regarding responding to early stroke symptoms, the EMS call option gathered the most preferences of the respondents by 80%. Enlightenment campaigns are needed to highlight acute stroke symptoms unknown to the general public such as facial asymmetry.

## Introduction and background

In 2019, acute ischemic stroke [1] was recorded as both a high risk of residual disability and high mortality worldwide. During the same year, acute stroke ranked second in deaths (11.6% of those who died overall), as well as third in the prevalence of death and disability combined [2] (5.7% of those who died and those who remained disabled combined) in the world. In addition, the burden of stroke has increased significantly.

Prior research reveals that the entirety of acute stroke-related deaths (86%) and disabilities (89%) [2] predominately occur in developing nations [3]. At the onset of the current decade (2020), the population of individuals with stroke-related disabilities witnessed a noteworthy surge, by 33.5 million, escalating from 91.5 million to 125 million.

In contrast, the administration of intravenous thrombolysis (IVT) for the treatment of cerebral ischemia has been shown to vertically improve the survival rate of patients and functional independence in activities of daily living. Because door-to-needle time (DNT) is vital in acute stroke effective treatment [4], administration of IVT requires that the time from the onset of symptoms to presentation to arrival at the emergency department (ED) does not exceed four to five hours. The efficacy-safety ratio of recanalization treatments such as IVT is time-dependent [1], so its supply be determined by strict limitations such as a short time window up to 4.5 hours. About 35% of patients are potentially eligible for IVT if targeted interventions such as calling emergency medical services (EMS) are achieved [5]. However, about 8-10% of them manage to receive IVT because the arrival time at the emergency department exceeds four to five hours [5].

Pre-attendance delays adversely affect the time window of recanalization treatments and the outcome of acute stroke [5-6]. Despite advances over the years, the time from symptom onset to arrival at the ED has remained unchanged over the past decade. Ignorance of recognizing stroke symptoms and seeking EMS are reversible factors, but consistently cause delayed arrival at ED [6] However, many people fail to understand that acute stroke is a medical emergency. Current implementation strategies do not contribute to the rapid reconnaissance of stroke symptoms and call of EMS to reduce delays and improve acute stroke outcomes [6]. 

Knowledge of the need for timely notification of EMS and transport by ambulance in acute stroke is correlated with reductions in the time interval between the onset of symptoms onset and the arrival at ED. The percentage of people who cannot recognize even one sign of face, arm, speech, and time (FAST) in the event of a stroke is estimated at 30.5% [6].

The overarching goal of this systematic review was to investigate the potential correlation between the population’s awareness of cerebral infarction symptoms, and their immediate activation through EMS, irrespective of sociodemographic factors or other hindrances to a timely and accurate response.

## Review

Methods

Study Design 

The researchers followed the statement of Preferred Reporting Items for Systematic Reviews and Meta-Analyses (PRISMA) [7], in the design and execution of this systematic review and took into account the guidelines for conducting and submitting a systematic review. The common practice among all authors is to research the most recent articles that should not be older than five years. The decision to focus on articles published from 2019 onwards was influenced by a preceding systematic review that had encompassed studies up to 2016 [8]. The aim was to conduct a synthesis of data from included studies to evaluate the likelihood of the entire community-dwelling adult population residing in the countries included in the study to recognize early signs, a process that affects the timely transfer to the ED and the onset of treatment. For the current review, a protocol has neither entered into a registry nor been prepared. 

Search Strategy and Eligibility Criteria

Between December 1, 2023, and February 29, 2024, a systematic review was performed using the PubMed database. Table 1 details the search strategies used to custom-filter the articles and the results. Two researchers were responsible for the search process until a review of available articles for inclusion or exclusion was completed.

**Table 1:** Search strategy starting from 2023/12/1 to 2024/02/29

Database	Search	Results
PUBMED	((“Knowledge”[MesH Terms] OR “Knowledge”[All Fields] OR “knowledge s”[All Fields] OR “knowledgeability”[All Fields] OR “knowledgeable”[All Fields] OR “knowledgeably”[All Fields] OR “knowledges”[All Fields] OR (“awareness”[MesH Terms] OR “awareness”[All Fields] OR “aware “[All Fields] OR “awarenesses”[All Fields])) AND (‘’people s”[All Fields] OR “peopled”[All Fields] OR “peopling”[All Fields] OR “persons”[MesH Terms] OR “persons”[All.Fields] OR “people”[All Fields] OR “peoples”[All Fields] OR (“populate"[All Fields] OR “populated”[All Fields] OR “populates”[All Fields] OR “populating”[All Fields] OR ”population”[MesH Terms] OR “population”[All Fields] OR “population groups”[MesH Terms] OR (“population”[All Fields] AND “groups”[All Fields]) OR “population groups”[All Fields] OR “populations”[All Fields] OR “population s”[All Fields] OR “populational”[All Fields] OR “populous”[All Fields])) AND (“diagnosis”[MesH Subheating] OR “diagnosis”[All Fields] OR “signs”[All Fields] OR “diagnosis”[MesH Terms] OR “diagnosis”[MesH Subheating] OR “diagnosis”[All Fields] OR “symptoms”[All Fields] OR “diagnosis”[MesH Terms] OR “symptom”[All Fields] OR “symptom s”[All Fields] OR “symptomes”[All Fields])) AND (“stroke”[MesH Terms] OR “stroke”[All Fields] OR “strokes”[All Fields] OR “stroke s”[All Fields])) NOT (“patient s”[All Fields] OR “patients”[MesH Terms] OR “patients”[All Fields] OR “patient”[All Fields] OR “patients s”[All Fields]))	The search in the PubMed database resulted in 1877 articles.
	((“Knowledge”[MesH Terms] OR “Knowledge”[All Fields] OR “knowledge s”[All Fields] OR “knowledgeability”[All Fields] OR “knowledgeable”[All Fields] OR “knowledgeably”[All Fields] OR “knowledges”[All Fields] OR (“awareness”[MesH Terms] OR “awareness”[All 4Fields] OR “aware “[All Fields] OR “awarenesses”[All Fields])) AND (‘’people s”[All Fields] OR “peopled”[All Fields] OR “peopling”[All Fields] OR “persons”[MesH Terms] OR “persons”[All Fields] OR “people”[All Fields] OR “peoples”[All Fields] OR (“populate"[All Fields] OR “populated”[All Fields] OR “populates”[All Fields] OR “populating”[All Fields] OR ”population”[MesH Terms] OR “population”[All Fields] OR “population groups”[MesH Terms] OR (“population”[All Fields] AND “groups”[All Fields]) OR “population groups”[All Fields] OR “populations”[All Fields] OR “population s”[All Fields] OR “populational”[All Fields] OR “populous”[All Fields])) AND (“diagnosis”[MesH Subheating] OR “diagnosis”[All Fields] OR “signs”[All Fields] OR “diagnosis”[MesH Terms] OR “diagnosis”[MesH Subheating] OR “diagnosis”[All Fields] OR “symptoms”[All Fields] OR “diagnosis”[MesH Terms] OR “symptom”[All Fields] OR “symptom s”[All Fields] OR “symptomes”[All Fields])) AND (“stroke”[MesH Terms] OR “stroke”[All Fields] OR “strokes”[All Fields] OR “stroke s”[All Fields])) NOT (“patient s”[All Fields] OR “patients”[MesH Terms] OR “patients”[All Fields] OR “patient”[All Fields] OR “patients s”[All Fields])) AND (y_5[Filter])	610 files left, after excluding 1267 records due to the use of the custom filter publication date before January, 1, 2019.
	((“Knowledge”[MesH Terms] OR “Knowledge”[All Fields] OR “knowledge s”[All Fields] OR “knowledgeability”[All Fields] OR “knowledgeable”[All Fields] OR “knowledgeably”[All Fields] OR “knowledges”[All Fields] OR (“awareness”[MesH Terms] OR “awareness”[All 4Fields] OR “aware “[All Fields] OR “awarenesses”[All Fields])) AND (‘’people s”[All Fields] OR “peopled”[All Fields] OR “peopling”[All Fields] OR “persons”[MesH Terms] OR “persons”[All Fields] OR “people”[All Fields] OR “peoples”[All Fields] OR (“populate"[All Fields] OR “populated”[All Fields] OR “populates”[All Fields] OR “populating”[All Fields] OR ”population”[MesH Terms] OR “population”[All Fields] OR “population groups”[MesH Terms] OR (“population”[All Fields] AND “groups”[All Fields]) OR “population groups”[All Fields] OR “populations”[All Fields] OR “population s”[All Fields] OR “populational”[All Fields] OR “populous”[All Fields])) AND (“diagnosis”[MesH Subheating] OR “diagnosis”[All Fields] OR “signs”[All Fields] OR “diagnosis”[MesH Terms] OR “diagnosis”[MesH Subheating] OR “diagnosis”[All Fields] OR “symptoms”[All Fields] OR “diagnosis”[MesH Terms] OR “symptom”[All Fields] OR “symptom s”[All Fields] OR “symptomes”[All Fields])) AND (“stroke”[MesH Terms] OR “stroke”[All Fields] OR “strokes”[All Fields] OR “stroke s”[All Fields])) NOT (“patient s”[All Fields] OR “patients”[MesH Terms] OR “patients”[All Fields] OR “patient”[All Fields] OR “patients s”[All Fields])) AND ((y_5[Filter]) AND (all adult[Filter]))	342 articles left after excluding 268 articles due to the use of the custom filter age.
	((“Knowledge”[MesH Terms] OR “Knowledge”[All Fields] OR “knowledge s”[All Fields] OR “knowledgeability”[All Fields] OR “knowledgeable”[All Fields] OR “knowledgeably”[All Fields] OR “knowledges”[All Fields] OR (“awareness”[MesH Terms] OR “awareness”[All 4Fields] OR “aware “[All Fields] OR “awarenesses”[All Fields])) AND (‘’people s”[All Fields] OR “peopled”[All Fields] OR “peopling”[All Fields] OR “persons”[MesH Terms] OR “persons”[All.Fields] OR “people”[All Fields] OR “peoples”[All Fields] OR (“populate"[All Fields] OR “populated”[All Fields] OR “populates”[All Fields] OR “populating”[All Fields] OR ”population”[MesH Terms] OR “population”[All Fields] OR “population groups”[MesH Terms] OR (“population”[All Fields] AND “groups”[All Fields]) OR “population groups”[All Fields] OR “populations”[All Fields] OR “population s”[All Fields] OR “populational”[All Fields] OR “populous”[All Fields])) AND (“diagnosis”[MesH Subheating] OR “diagnosis”[All Fields] OR “signs”[All Fields] OR “diagnosis”[MesH Terms] OR “diagnosis”[MesH Subheating] OR “diagnosis”[All Fields] OR “symptoms”[All Fields] OR “diagnosis”[MesH Terms] OR “symptom”[All Fields] OR “symptom s”[All Fields] OR “symptomes”[All Fields])) AND (“stroke”[MesH Terms] OR “stroke”[All Fields] OR “strokes”[All Fields] OR “stroke s”[All Fields])) NOT (“patient s”[All Fields] OR “patients”[MesH Terms] OR “patients”[All Fields] OR “patient”[All Fields] OR “patients s”[All Fields]) AND ((y_5[Filter]) AND (english[Filter]) AND (all adult[Filter])).	After excluding 2 articles due to the use of the custom filter article language 340 articles left which screened and assessed for eligibility.

Articles were incorporated if they met the inclusion criteria listed below, or were excluded if they met at least one of the exclusion criteria (Table 2). To compile the inclusion and exclusion criteria, the two researchers of the current review who conducted the PubMed database search took into account the population, intervention, comparison, outcome measures, and study design (PICOS) model [9].

**Table 2:** Population, intervention, comparison, outcome measures, study design (PICOS) criteria

PICOS ITEM	INCLUSION CRITERIA	EXCLUSION CRITERIA
Population	People without cerebrovascular or cardiovascular history, healthy or diseased individuals.	People who have suffered from cerebrovascular or cardiovascular disease.
Intervention	The research of global awareness and response to early symptoms of acute ischemic stroke.	Failure to research global awareness and response to early symptoms of acute ischemic stroke or a sub-topic such as global awareness.
Comparison	Contained data on the ability of the general population to detect early symptoms and respond to acute ischemic stroke.	Did not include in the title or abstract research on the general population's awareness of early symptoms and response to acute ischemic stroke.
Outcome measures	Correlation between sociodemographic characteristics, global awareness, and response to early symptoms of acute stroke Measured outcomes of interest for the current review and were relevant to the study objectives.	Measuring only awareness of one or all early symptoms or only the correct response or not to the acute stroke or neither, or without association with sociodemographic factors Studies in which the results did not follow the inclusion criteria or were insignificant or unclear.
Study Design	Cross-sectional studies.	Research protocols, systematic reviews, meta-analyses, letters, case reports, and narrative reviews.
Publication	Published in English-language peer-reviewed journals, published between 1 January 2019 and 31 December 2023, open access to full text.	Published in non-English-language journals, or non-peer-reviewed journals, not available in full text.

The two researchers independently reviewed the titles and abstracts and followed up with a thorough analysis of the full text of the articles according to the predefined inclusion criteria. In cases where a discrepancy arose between the two researchers’ assessments and a consensus could not be reached, a third senior researcher intervened to reconcile the differing opinions. The inter-rater agreement was nearly perfect and equal, reaching 90% for both study selection and data extraction.

The primary outcomes of the included studies contain data on participants' sociodemographic characteristics, in the order of age group, gender, race, ethnic group, marital status, professional skills, monthly income, place of living, and chronic diseases.

The assessment of awareness of early symptoms of acute ischemic stroke and the early activation with EMS notification belong to the secondary outcome measures of the studies in the present review.

Data Extraction and Interpretation

The following data were extracted by the researchers: (i) corresponding author, (ii) article publication date, (iii) the type and size of the sample, iv) the sampling method, v) the selection process of the subjects, vi) sociodemographic characteristics of participators including age diversity of the target population, level of education, income bracket, professional position, geographic origin, and medical history vii) the awareness of early symptoms of acute ischemic stroke, viii) the response to early symptoms of stroke, and ix) the impact of sociodemographic characteristics in awareness of early symptoms and the response to early symptoms. We followed this specific methodology to compare studies and extract data clearly and accurately. 

Risk of Bias

The review researchers used the critical assessment tool for cross-sectional studies - appraisal tool for cross-sectoral studies (AXIS) [10], to evaluate the quality of the comprised articles. It is a twenty-question integrated tool that systematically evaluates, judges the reliability and validity, and assesses the value and relevance of the included studies. The questions concern the introduction, the objectives, the representation of the general population in the selected sample, the methods, the results, the discussion, and other issues of conducting the studies. Since the tool has assumed popular consent among health professionals and the academic community, as well as having a high level of consensus among researchers, it has also been approved for use in systematic reviews [10]. Furthermore, the studies included in this systematic review were entirely cross-sectional, so it was considered a tool to be implemented. Therefore, a descriptive formulation of the results was performed by summarizing the results of the individual studies. The results of each study contain different knowledge of early symptoms and awareness of participants according to p values, confidence intervals, odds ratios, and standard errors. 

Results 

**Figure 1:**
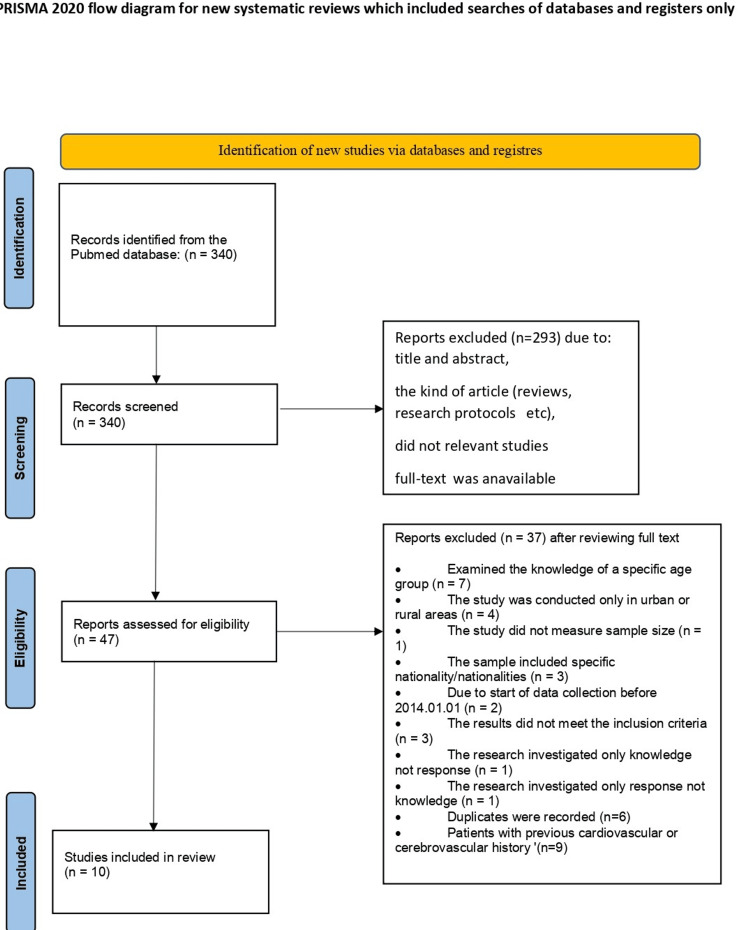
PRISMA study flow diagram for systematic review PRISMA: Preferred Reporting Items for Systematic Reviews and Meta-Analyses.

The database search resulted in 340 records with the application of custom filters. After the 340 articles were screened, 293 reports were excluded due to title and abstract, because they were not relevant studies or where the full text was not available. Following the application of inclusion criteria, the articles were narrowed down to 47 and subjected to further analysis and refinement (Figure 1). 

Out of these records, ten met the inclusion criteria. The details of the included studies are presented in Table 3.

**Table 3:** Presentation of the details of included studies

First author’s Name Date of Publication Country	Type Of studies: Cross-sectional / Data collection tool	Population	Sample size- Νumber of respondents	(Response rate %)	Participant selection.	Population age/min-max limit (years)	Women’s number in the population sample.
Barakat et al. [11] , (2022), Jordan	Online Questionnaire	Jordanian population of all regions 18 years and older.	573/573	100%	Snowball sampling method	18-70+years	373 (65.1%)
Krishnamurthi et al. [12] (2020), New Zealand	Telephone Interview	Adults aged twenty and over from across New Zealand.	400/228	57%	Stratified random sampling	20-60+ years	200 (50%)
Navia et al.[13],^(^2022), Chile	Telephone Interview	Metropolitan or non-metropolitan residents of all regions of Chile aged 18 or over, women and men.	3,362/706	22%	Stratified random sampling	18-55+ years	354 (50.1%)
Han et al. [14], (2019), Κorea	Face-to-face Interview	Nationwide study subjects 19-110 years old.	228.381/ 228.240	99.9%	Two-stage systematic random sampling	19-110 years	125.832 (55.13%)
Luan [15], 2021, China	Face-to-face Interview	Structured household survey of four major economic regions of Mainland China.	3,051/1,525	50%	Simple random sampling	18-69 years	1,473 (48.3%)
Jirjees et al. [16], (2023), United Arab Emirates (UAE)	Online Questionnaire	Public general groups over 18 years in the United Arab Emirates.	593/545	92%	Snowball sampling method	18-50+ years	319 (58.5%)
Oh et al. [17],(2019), Korea	Face-to-face interview	individuals ≥ 19 years of age in the Metropolitan area of Jeonbuk.	12,428/ 10,445	84%	Unclear	19-70+ years	5,954 (57%)
Patel et al. [18], (2019), USA.	No clear description of the data collection tool	Civilian non-institutionalized population ≥ 18.	63,073/ 63,073	100%	Multistage random sampling	18-65+ years	35.043 (51.55%)
Oh et al. [19],(2022) Korea	Face-to-face interview	Members of households older than 19 years old.	228.381/ 198.403	86.90%	Two-stage systematic random sampling	19-90+ years	107.585 (54.2%)
Zafar et al. [20], (2020) Saudi Arabia.	Online Questionnaire	Eastern Province Inhabitants of Saudi Arabia ≥15.	1,213/1,213	100%	Unclear	15-60+	757 (62.4%)

Main Characteristics of Included Studies

The ten included studies [11-20] were published between January 1, 2019, and October 11, 2023 (Table 3). Four of the studies [11, 13, 15-16], were carried out between January 1, 2019, and November 30, 2021, while the remaining six studies [12, 14, 16-20], were conducted between January 1, 2014, and December 31, 2018. Out of ten studies, one study [11] was conducted in Jordan, one study [12] was conducted in New Zealand, the country of origin for the study [13] is Chile, while in the studies [14, 17, 19] the corresponding author is frοm South Korea. There is one Chinese study [15], one study is from the United Arab Emirates [16], and another one was conducted in the USA [18]. The country of origin for the study [20] is Saudi Arabia.

All studies were designed and conducted as cross-sectional studies. Out of the ten studies of the current review, one was conducted as a descriptive cross-sectional study [11], six as a cross-sectional study [12-15, 17, 19], one as a cross-sectional observational study [16], one as a national health interview study [18], and the last one as a prospective cross-sectional study [20]. Online questionnaires were used as data collection tools in three studies [11, 16, 20], face-to-face interviews in four studies [14-15, 17, 19], while telephone interviews were used to obtain data in two studies [12-13], and in one study [18], the data collection tool is not accurately described. Three of the studies did not specify exactly how long the investigation lasted [14, 17-18].

The total sample in the ten studies amounted to 504,951 people. Based on each study, the numerical range of respondents was from 400 [12] to 228,240 [14]. In two studies [11, 16], subjects were chosen through the snowball sampling method, while in another two studies, [12-13], stratified random sampling was employed. One study [15], utilized simple random sampling, and two studies [14, 19], utilized a two-stage systematic random sampling approach. Both studies [17, 20], did not provide clear and precise definitions for the sampling methods applied in their studies. Another study [18], detailed a multistage sampling approach. In all studies, the respondent group included only adults, except for one study [20], in which, in addition to adults, minors aged 15-17 years old were included as well.

As the measurement of awareness of early symptoms and response to acute stroke were very different and in many studies, the sample characteristics were not correlated with each other, it was not possible to include the data in a meta-analysis.

Risk of Bias in Included Studies

The two researchers, responsible for data collection and processing, assessed studies meeting the inclusion criteria using AXIS [8], a critical appraisal tool for cross-sectional studies. The evaluation results are documented in Table 4. Through this tool, researchers observed variations among studies in terms of quality, sampling methods, and types of errors.

**Table 4:** Controlling risk of bias in included studies by applying the critical appraisal tool AXIS Tool for cross-sectional studies. CI: confidence interval, AXIS: appraisal tool for cross-sectoral studies.

FIRST AUTHOR	INTRODUCTION: 1) Were the aims/ objectives of the study clear?	METHODS: 2) Was the study design appropriate for the stated aim(s)?	3) Was the sample size justified?	4) Was the target/reference population clearly defined? (Is it clear who the research was about?	5) Was the sample frame taken from an appropriate population base so that it closely represented the target/reference population under investigation?	6) Was the selection process likely to select /subjects/participants that were representative of the target/reference population under investigation?	7) Were measures undertaken to address and categorize non-responders?	8) Were the risk factors and outcome variables measured appropriate to the aims?	9) Were the risk factor and outcome variables measured correctly using instruments /measurements that had been trialed, piloted, or published previously?	10) Is it clear what was used to determine statistical significance and/or precision estimates? (e.g. p values, CIs)	11) Were the methods (including statistical methods) sufficiently described to enable them to be repeated?	RESULTS: 12) Were the basic data adequately described?	13) Does the response rate raise concerns about non-response bias?	14) If appropriate, was information about non-responders described?	15) Were the results internally consistent?	16) Were the results for all the analyses described in the methods presented?	DISCUSSION: 17) Were the authors' discussions and conclusions justified by the results?	18) Were the limitations of the study discussed?	OTHER: 19) Were there any funding sources or conflicts of interest that may affect the authors' interpretation of the results?	20) Was ethical approval or consent of participants attained?
Barakat et al. [11]	Y	Y	Υ	Υ	Y	Ν	N	Υ	Y	Y	Y	Y	N	NA	Y	Y	Y	Y	N	Y
Krishnamurthi et al. [12]	Y	Y	Ν	Υ	Υ	Υ	Y	Υ	Y	Y	Y	Y	Y	Υ	N	Y	Y	Y	N	Y
Navia et al. [13]	Y	Y	Y	Υ	Y	Υ	Ν	Υ	Y	Y	Y	Y	Y	Y	Ν	Y	Y	Y	N	Y
Han et al. [14]	Υ	Y	N	Υ	Υ	Υ	Ν	Υ	Y	Y	Y	Y	N	NA	Y	Y	Y	Y	N	Y
Luan et al. [15]	Υ	Y	Y	Υ	Υ	Υ	Y	Υ	Y	Y	Y	Y	N	NA	Y	Y	Y	Y	N	NN
Jirjees et al. [16]	Υ	Y	Y	Υ	Y	Ν	Ν	Υ	Y	Y	Y	Y	N	NA	Ν	Y	Y	Y	N	Y
Oh et al. [17]	Υ	Y	N	Υ	Υ	Ν	Ν	Υ	Y	Y	Y	Y	N	NA	Υ	Y	Y	Y	N	Y
Patel et al. [18]	Υ	Y	N	Υ	Υ	Υ	Ν	Υ	Y	Y	Y	Y	N	NA	Υ	Y	Y	Y	N	NN
Oh et al. [19]	Y	Y	N	Υ	Y	Υ	Υ	Υ	Y	Y	Y	Y	N	NA	Y	Y	Y	Y	N	Y
Zafar et al. [20]	Υ	Υ	N	Υ	Y	Ν	Ν	Υ	Y	Y	Y	Y	N	NA	Y	Y	Y	Y	N	Y

The sample size was justified in four studies [11, 13-15]. The sample frame in all studies [11-20], was drawn from an appropriate population base to closely represent the target/reference population under investigation. Seven of the studies [10-13, 15, 17], recruited subjects, who were representative of the target/reference population under investigation. Α similar selection process did not occur for the selection of the subjects in three studies [9, 14, 18], therefore the selected subjects did not represent the population under investigation. Three studies [11, 13, 17], took steps to address and categorize non-responders if the participation rate was insufficient. In all ten articles included in the review, not only were early symptoms (risk factors excluded) and response to acute stroke measured but also the outcome variables appropriate to the study objectives.

Furthermore, all studies in this review [11-20], used measures/instruments that had been pilot-tested or previously published and were clear about the methodology used to determine statistical significance and/or precision estimates. All ten studies [11-20], described the methods they followed in sufficient detail to allow replication. In all included articles, baseline data were equally described.

However, two studies [10-11], have a response rate that raises concerns about non-response bias and provides information about non-responders. In the remaining studies, this was not applicable as the response rate was either complete (100%) in some studies [9, 12, 16, 18] or high in others [13-15, 17]. Internal consistency distinguishes the results of seven studies [9, 12-13, 15-18], except the results of the three remaining studies [10-11, 14]. The results of all ten studies were analyzed using the procedures outlined in the methods. The authors' conclusions and discussions were validated with these results [8]. A key feature of the studies in this review is the discussion of limitations in all of them. Neither conflict of interest nor any source of funding was considered capable of influencing the interpretation of the results by the authors in any of the studies.

Only one study [17], did not report whether or not there was a conflict of interest. Nonetheless, such a possibility is removed as it did not receive any specific grant. In four studies [9, 15, 17-18], ethical approval or participant consent was given, while the investigators of the studies [11, 17], secured approval in addition to consent. In the remaining three, [12-13, 16], it was not deemed necessary to obtain approval or consent because all data were de-identified.

Results of Each Study Separately 

Individually examining each study, it was observed that the early symptoms frequently recognized in acute stroke included difficulty in speaking or understanding speech, weakness on one side of the body (including the face), and sudden dizziness (Table 5). Among the ten studies reviewed, nine [11, 13-20], asked closed-ended questions (prompted methods), about awareness and response to acute stroke, with possible answers of yes, no, or I don't know. In contrast one study [12], utilized both closed and open-ended questions, (unprompted methods), with the most frequently recognized symptoms including difficulties in speaking or understanding speech, weakness on one side of the body (including the face), and sudden double or blurred vision.

**Table 5:** Details of results of each study separately SWS: stroke warning symptoms, FAST: face, arm, speech, and time, OR: odds ratio, CI: confidence interval, SES: socioeconomic status

First author (Year) Country	OPEN OR CLOSED ENDED QUESTIONS?	Knowledge of early symptoms of stroke	Awareness of response to acute stroke
Barakat et al. [11], (2022), Jordan	Closed-ended	The most recognizable SWS were found: i) Sudden difficulty in speech, or understanding speech 92.3% ii) Sudden weakness,/numbness,/tingling 88% recognition of at least one symptom of stroke i) university level 94% ii) school level 6% i) participants with no history of diabetes 92.3% ii) participants with a history of diabetes 7.7% Recalling at least one stroke symptom 95.5%.	Attitude and reactions towards stroke: Taking a patient to the hospital: among all participants 89% i) university level 94.3% vs. Scholar level 5.7% ii) employed 62.2% vs unemployed-37.8% iii) participants with no history of diabetes 92.7% vs participants with a history of diabetes 7.3%.
Krishnamurthi et al. [12], (2020), New Zealand	Unprompted (open-ended) and prompted) (close-ended) methods of questioning were utilized	Recognition of stroke symptoms was assessed using only a prompted method The most commonly identified symptoms were: i) Sudden speech difficulty 94% ii) Sudden weakness on 1 side 92% 98% of participants identified 1 stroke symptom 96% identified 2 or more, 86% identified 3 or more.	Response to the question “If you or someone you know has any symptoms of a stroke what should you do? i) Call the ambulance or emergency services 80% of the participants ii) visit or call their general practitioner or manage the condition themselves 20% of the participants.
Navia et al. [13], (2022), Chile	Closed-ended	Regarding recognizing stroke symptoms: 74.4% of the participants recognized at least one typical stroke symptom and 6.6% recognized the 3 symptoms of the FAST scale. (speech difficulty, weakness of one arm, or leg, and face dropping) I) Headache was the most frequently selected symptom 44.2% ii) Speech difficulty 43.8% iii) Weakness of one arm, or leg 38.1% iv) Face dropping 36.5% participant characteristics associated with less recognition of at least one stroke symptom: men (OR O.67 95% CI 0.47-0.96) 55 years old, or older (OR 0.59 95% CI 0.37-0.93) vs younger group being in the lowest SES (OR 0.33 95% CI 0.6-0.67), vs being in the wealthier group.	In case of a suspected stroke: Most of the participants 82.4% chose to go directly to the hospital emergency care or contact the mobile emergency service or another ambulance service Participants who recognized at least one typical stroke symptom and they attended an emergency service in case of stroke were: i) 66%, 95% CI 60,1-71.3 18-34 years old ii) 64%, 95% CI 57.8-69.2% 35-54 years old iii) 52%, 95% CI 44.5-59.7% ≥55 years old iv) 75%, 95% CI 66-82 upper middle SES v) 60%, 95% CI 53-67 middle SES vi) 63%, 95% CI 56-70 lower middle SES vii) 62%, 95% CI 54-69 lower SES viii) 40%, 95% CI 29-53 lowest SES
Han et al. [14], (2019), Korea	Closed-ended	Knowledge about symptoms of stroke: i) Sudden numbness or weakness in the face arm or leg 75.7% ii) Sudden confusion, trouble speaking or understanding others 80.4% iii) Sudden poor vision in one or both eyes 66.1% v) Sudden dizziness difficulty walking or loss of balance 77.3% vi) Sudden headache with no known cause 66.5%. Women had better knowledge of stroke symptoms than men (p<0.0001). The least identified stroke symptom was sudden blindness in one or both eyes 66.1%.	Of all the respondents 79.4% would call an ambulance when someone had symptoms or signs of stroke.
Luan et al. [15],(2021), China	Closed-ended	Participant’s recognition of stroke symptoms: numbness 67.6% lopsided face 66.7% slurred speech 65.2% spit running out of the mouth 64.2% paralysis 52.6% having problems eating 24.7% difficulties swallowing 23.3% one-sided blindness 21.3% runny eyes 14.4% sudden confusion 11.5% experiencing a prickly feeling 9.9% Over 92% of the participants recognized at least one of the three symptoms mentioned in the FAST test. A small percentage incorrectly thought that earache was a symptom of stroke (6.5%) On average participants recognized 5,2 (SE =0.05) out of 14 symptoms.	First reaction at the onset of stroke: 75.9% would call an ambulance 58.8% advise the person to see a doctor 34.1% advise the person to consult a doctor immediately 1/3 would recommend bedrest 1/10 would suggest sipping fluid 66% among participants who recognized none of the three symptoms would call an ambulance 70% who recognized at least one 75% who recognized at least two 82% who recognized all three symptoms With regard to calling an ambulance, was found that with each additional symptom recognised, the probability of calling an ambulance increased by two percentage points.
Jirjees et al. [16], (2023), United Arab Emirates (UAE).	Closed-ended	Recognition of early stroke symptoms: Only 21,3% of participants were able to recognize all symptoms of a stroke. Symptoms most frequently reported by participants: i)sudden difficulty speaking/ understanding speech ii) 78%, loss of consciousness/fainting iii) 73.8% iv) sudden dizziness 71,4%. Correct identification of stroke symptoms i) females (92.8%vs males 85,8%, p= (0,008) (OR 1,9) ii) university level 92,8% vs scholar level 82,9%, p= (0,001). (OR 2,5 ) medium income (OR 3,1) vs low income (OR 2,5 ) just over 20% of the participants recognized all of the symptoms recalling at least one stroke symptom 89.9% of participants.	Taking the patient to the hospital smokers (OR Of 1,5) vs non-smokers 23.9% of the participants were encouraged to go to a hospital as soon as possible after a stroke was detected
Oh et al. [17], (2019), Korea	Closed-ended	Individual early stroke symptoms and recognition: by all sampled employees 75.5% for sudden difficulty speaking or understanding speech 75.2% for sudden dizziness 73.3% for sudden numbness or weakness 63.6% for sudden severe headache 62.2% for sudden visual impairment number of SWS correctly identified among the participants 12.7% identified none SWS correctly 5.0% identified one SWS correctly 8.5% identified two SWS correctly 11.9% identified three SWS correctly 17.5% identified four SWS correctly 44.4% identified five SWS correctly.	Association of occupation with awareness of early symptoms and emergency response to stroke Odds ratio and 95% confidence interval MODEL 1 UNADJUSTED MODEL 2 MODEL 3 (REFFERENCE) Managers and 1 Professionals * (REFFERENCE) 1 (REFFERENCE) 1 Clerks 1.09 1.06 1.05 Service and sales workers 1,09 1.14 1.15 Agricultural, forestry 1.14 and fishery workers 1.42 1.30 Mechanical and manual 0,99 laborers 1.07 1.12 Housewives and unemployed 0,84 people 1.16 1.18 *Benchmark Managers and Professionals adjusted for age and gender-adjusted for gender age residence type marital status educational level monthly household income.
Patel et al. [18], (2019), USA	Closed-ended	Prevalence of stroke symptom awareness Numbness of face, arm, leg, side 92% Confusion, trouble speaking 91,5% Trouble walking 90% Sudden trouble seeing 82% Sudden severe headache 77%.	Awareness of the importance of calling 911 96% of individuals Knew to call 911 if someone was having a stroke 67% of individuals had knowledge of all five stroke symptoms and Knew the importance of calling 911 or other emergency numbers.
Oh et al. [19], (2022), Korea	Closed-ended	Percentage of correct answers to SWS 4-5 answers: i) 66.5 % defined of all study subjects ii) 72.6% from middle-aged group iii) 63.5% from young group iv) 61.5% from older group v) The greatest knowledge was shown in those in their 50s 73% The most recognizable of the early symptoms were the young adults middle-aged adults older adults. Sudden difficulty in speaking or trouble understanding speech 79.1% 86.1% 77.2%; Sudden dizziness or loss of balance 76.4% 82.5% 74.5%; Sudden unilateral weaknesses of face arm or leg 72.2% 81.3% 75.5%; Sudden severe headache 67.2% 71.6% 62.8%; Sudden visual impairment in one eye or double vision 64.4% 72.6% 63.3%.	Call an ambulance young adults 79.4%, middle-aged adults 81.3%, older adults 78.3%.
Zafar et al. [20], (2020), Saudi Arabia	Closed-ended	Knowledge of respondents about signs of stroke speech difficulty 64.4%, focal weakness 62.2%, loss of balance 44.3%, change in consciousness 43.8%, facial asymmetry 40.1%, vertigo 26.9%, visual impairment 22.1%, headache 23.5%, double vision 16.8%.	Awareness about stroke treatment contact with a personal doctor or family 10.4%, take aspirin directly and wait until symptoms improve 5.8%, massage circularly with hot compresses until symptoms improve 3.2%, take rest and sleep until symptoms improve 0.5% contact the ambulance directly and transfer the patient to the nearest hospital 80.3%.

The nine studies employing closed-ended questions reported a percentage of the sample correctly identifying at least one early symptom of acute stroke ranging from, 74.4% [13], to 95.5% [11]. In another study, 98% of participants were capable of correctly identifying an early symptom of acute stroke [12].

All seven of the highest quality studies in the current review asked closed-ended questions about early symptoms and response to stroke, concluding that among respondents 81.5% could correctly identify at least one early sign of acute stroke [11, 14-15, 17-20].

Regarding the association of responses with age, an increased level of awareness and correct response to early symptoms of acute stroke was found in the middle age category of ≤ 60 years old in the Chinese study [15], while the age limit varied between 50 and 69 years in the first Korean study [14]. Furthermore, people who responded with correct answers were between 45 and 65 years old in the third Korean study, [19]. On the contrary, similar findings were reported in the USA study [18], in the age group of 18-44 years.

Among all studies, studies [11, 14-15, 17, 19], were identified as correlating awareness of early symptoms and response to acute stroke with both a high level of education and a high income.

Four studies indicated a prevalence of women in their responses, in terms of gender [11, 14-15, 17].

In terms of the clinical indicators, the existence of a medical history did not seem to have a clear positive or negative effect on the outcome. In particular, a medical history was combined with either positive answers [15, 17-18], or negative ones [14], but in others [11-13, 17, 20], appeared to be an independent factor. Concerning the response to early stroke symptoms, participants reported either immediately calling EMS or directly transporting the patient to the ED by private vehicle. Immediate calling of (EMS) or other types of ambulance services was considered to be the most appropriate response ranging from, a minimum of 75.9% [15], to a maximum of 95% [18]. One study [11], did not provide exact percentages for each option, whereas another [17], did not present percentage results whatsoever.

In the study [19] 80% of the sample utilized the three-digit health emergency number, less than in a USA study [21], but more than in a corresponding Korean study [22]. Οnly 66% of those who did not recognize any of the three FAST symptoms, called for an ambulance, compared with 70% who recognized at least one, 75% who recognized at least two, and 82% who recognized all symptoms [15]. Early symptom recognition in acute stroke does not always translate into action due to the reluctance of those involved to notify EMS. The reasons why subjects did not choose a rapid (EMS) notification due to a lack of understanding of the severity of the condition, or the expectation of remedying the condition without the assistance of health professionals. Another cause of delay was the decision to transfer the affected person to the ED by another type of vehicle [23].

At the onset of acute stroke, the presence of serious symptoms has a positive effect on both seeking help rapidly and EMS notification [24], as does cohabitation with a partner.

Although 80.3% of respondents chose to notify the ambulance services, more than 33% of subjects failed to distinguish between slurred speech and focal weakness while more than 50% ignored facial asymmetry as an early symptom of acute stroke [20].

Risk of Bias in Correlation to Study Findings

Although the AXIS critical appraisal tool [10] does not perform quantitative measures to assess the quality of studies, in some studies the participators were found to give more positive answers to the questions [11, 14-15, 17-20] and were therefore considered to be more dependable. The aforementioned studies demonstrated the association of higher education level with a greater depth of knowledge which leads to a correct response to the early symptoms of acute stroke.

Two studies [12-13], of the three remaining with lower response scores on the AXIS, showcased a deeper knowledge of early acute stroke symptoms and an awareness to respond to stroke by individuals with higher levels of education and income.

Furthermore, in the third study [16], more positive answers were recorded when women, were asked as well.

Discussion

To the best of the authors’ knowledge, this is the first systematic review to provide worldwide information, incorporating the most recent data, not only on the general population’s awareness of early symptoms but also on their response to the onset of acute stroke.

Αwareness of Early Symptoms and Correct Response to Acute Stroke Among Participants

Of the seven studies that were found with internal consistency [11, 14-15, 17-20], both higher education and a higher income were demonstrated to be a main feature of an individual’s awareness of early symptoms and correct response to acute stroke. Among high-income and highly educated participants, women, [11, 14-15, 20], those with a prior medical history, [14-15, 17, 19], and those living in urban areas [11, 14-15], indicated increased awareness and correct response. Residents of urban areas have better access to information resources and health care than those in rural ones. The only exception was one study [18], reporting better findings among subjects who combined both lower levels of education and lower income. This study was compared to a corresponding study in 2009 in the same country. In one study [20], the association between sociodemographic and clinical characteristics and outcomes remained unclear.

Best Findings Among Included Studies of This Review Comparing With Corresponding Studies

The best recognition rates of early symptoms of acute stroke compared to other population-based studies originating from the European area [25], and almost the same as the recognition rates in Germany, which presented the best findings among the rest of including studied countries, were presented by the Chinese study [15]. However, in relation to more recent rates of research remain lower compared to those of the Singapore study and the South Korean study [26-27]. Among respondents 92% identified at least one of the three symptoms included in the acronym FAST.

Priorities and Target of Informational Programs and Mainstay of Policy

Exploring knowledge of early symptoms and awareness of response to acute stroke revealed a social dimension of the findings of the present systematic review. Males, the elderly, individuals who studied up to secondary education, live alone, without employment, on the verge of poverty, with a history of hypertension or dyslipidemia, all of the above indicators predispose to a lack of knowledge and correct response to acute stroke [7, 14]. Not only understanding but also increasing the citizens' awareness belonging to a low socio-economic class may be always the mainstay of policy to prevent stroke and to limit the consequences of the appearance of the disease. [7, 14, 19]. Beyond this, the need to implement informational programs regarding the signs and symptoms of stroke in targeted populations is evident [13-14, 19].

A large-scale Korean study [14], reported that a four-month educational program of elderly people, with an average age of 75 years old, improved the subject’s ability to recognize symptoms of cardiovascular disease.

Strengths and Limitations of the Review

Our review could not avoid some limitations, one of them being that the authors were unable to perform a meta-analysis. Heterogeneity of the sociodemographic characteristics of the samples was evident in many of the included studies. Regarding the correlation of answers with age, differences were found in terms of correct answers in the upper and lower age limits even among people of the same age group (middle age) in studies [14-15, 19]. In contrast in the study [18], a higher percentage of positive responses was found in a different age group (young age). Furthermore, a significant variable regarding residence in an urban or rural area was identified in only three studies [11, 14, 19].

Knowledge of early symptoms and response to acute stroke was recorded using the prompted method (closed-ended questions) but this may have inflated the rate of awareness and correct response. By using open-ended questions researchers are essentially biasing respondents by directing their choice to the desired answer. The risk of bias such as sampling bias became visible as not all studies had a sufficient sample size so the number of participants in each study was representative of the general population (Table 4).

Some studies were unable to avoid systematic information bias due to the data collection tools (online surveys or telephones) they chose. Not only could a great number of respondents ensure availability and access to the data collection tools during the conduct of the studies, but they did not have a sufficient level of education to operate them either. Many people of low socio-economic status lacked a telephone. Our search did not yield interesting current studies from the European Continent, however, the fact that stroke is the second most fatal disease as well as the third most common disease in prevalence which can cause either disability or death worldwide, is a reason to update the findings in Europe as well. These findings are in line with a previous review [2].

In relation to the results of a former review [8], the current findings add new information, due to correlated participants' occupation and awareness with response to early symptoms of acute stroke. An advantage of this review is for the first time the knowledge of early symptoms and awareness by the general population worldwide to respond when an acute stroke occurs has been investigated. The most recent information including factors such as age group, presence or absence of medical history, and cohabitation with a spouse or partner have also been incorporated. What is more, participants’ level of education determined their economic and social status in all studies as well as the extent of knowledge and the type of response. Additionally, compared with a previous systematic review [8], the current review innovates by introducing data regarding the effect of socioeconomic status in rapid correct response to an acute stroke.

An intervention to stress, the value of awareness of the early signs, and response between the onset of a stroke and notification of EMS, the FAST test, can simplify the process of recognizing acute stroke, which is presented with symptoms variations but which also requires combinatorial knowledge to be detected.

What is also important is the fact that both the recognition and learning of the details of early stroke symptoms, when an acute stroke occurs, can be understood without much effort on the part of the general public. Compared with other prehospital stroke scales both acute sensitivity and moderate specificity are discerned, regardless of severity and age [15, 28].

Increased trends were observed in all acute stroke patients treated with IVT by a group of Norwegian researchers, after promoting a strategy through mass media, which introduced the teaching of the FAST acronym to the community [20, 29]. In addition, Canadian Stroke Best Practices has suggested the design of educational campaigns based on the FAST method for the recognition of acute stroke [30].

Although the (FAST) method detects an increased number of acute strokes, a significant percentage of posterior circulation strokes may be overlooked, such as visual symptoms and balance disorders. In relation to the preview review [9], which did not indicate a corresponding tool such as an acronym or mnemonic, the authors of this systematic review suggest the universal application of balance, eye, face, arm, speech, time (BE FAST) mnemonic. This proposal is in line with studies [17, 31-32]. It could contribute to early recognition of > 95% of acute strokes [32]. Utilizing the FAST Test it is possible for some patients, mainly in early or middle age with fewer severe deficits not to be detected [32]. No gender-attributable differences are identified in the assessment. However, gait impairment or sight loss as recognized by the mnemonic BE FAST tends to cause severe disabilities requiring reperfusion therapies.

Implications for Implementation

Future researchers into acute stroke are encouraged to determine exactly what the impact of the knowledge of early symptoms and awareness of rapid response is. Policymakers, decision-makers, and healthcare providers need to determine how many patients, after the onset of acute stroke, might have avoided side effects, such as life-long disabilities or even death. It would be essential for policymakers, decision-makers, and healthcare providers to determine how many patients, after the onset of acute stroke might have avoided side effects such as long-life disabilities or even death. How does the knowledge of early symptoms, and the awareness of acute stroke, after the immediate notification of EMS, influence the rate of deaths and disabilities? Moreover, primary healthcare professionals could utilize both digital health records and teamwork to undertake the educational process [2]. Public education campaigns must emphasize the phrase “time is brain” [23] so that acute stroke can be understood as a medical emergency. Prehospital mobilization, concerning acute stroke, through early notification of EMS is vital to increase stroke patients’ timely access to primary stroke centers, to receive IVT, and avoid side effects such as long-term disability [2]. Although it is considered a prudent solution for a bystander to transfer a patient to the hospital, it is recommended and preferable for a patient with the first symptoms of an acute stroke to contact EMS. After heeding patients’ or their bystanders’ responsiveness, the outcome of the patient is likely to be improved if, within 4.5 hours of acute stroke onset they are given access to the relevant healthcare providers [5, 13-14, 17]. Otherwise, they might be able to assist emergency department staff with their valuable information to be classified as severely affected patients who need to benefit from carotid endarterectomy if they are immediately transferred to cross-disciplinary stroke centers.

## Conclusions

As a result, the present article reports on a novel, more effective tool to conduct educational campaigns or measurements of awareness and response to acute stroke, the BE FAST mnemonic. Using this aid, the general public is likely to become aware of early symptoms whilst simultaneously gaining a deeper understanding of the mechanism by which acute stroke occurs. The correct recognition of the early signs has proven to be critical regarding the awareness of early mobilization through notification of EMS as all studies of the current review have shown. The level of knowledge of the early symptoms and the response of the general population to acute stroke remains unclear as long as researchers follow inductive methods with closed questions. Open-ended questions more accurately measure the level of knowledge and objectively reveal the need for public enlightenment. Both global awareness of response to early symptoms of acute stroke invariably remain at low levels, due to acute stroke being ranked second in deaths, as well as third in the prevalence of death and disability combined. Therefore, based on the above information it could be exacted that the enlightenment efforts fail to have a long-lasting effect and fail to be recalled to memory.
